# Supplementary material for: Heat adaptation of phage T7 under an extended genetic code
Source: Virus Evol. 2021 Dec 1;7(2):veab100. doi: 10.1093/ve/veab100 (PMC8923235; doi:10.1093/ve/veab100)
Supplement: veab100_Supp [file veab100_supp.zip › Sequencing_Analysis.zip!/Sequencing_Analysis/A3/evidence/SNP_14.html]

BRESEQ :: Evidence


| Predicted mutation | | | | | | |
| --- | --- | --- | --- | --- | --- | --- |
| evidence | seq id | position | mutation | annotation | gene | description |
| RA | NC\_001604.1 | 35,095 | A→G | intergenic (–/–) | – / – | –/– |

| Read alignment evidence... | | | | | | | | | | | |
| --- | --- | --- | --- | --- | --- | --- | --- | --- | --- | --- | --- |
|  | seq id | position | | ref | new | freq | score (cons/poly) | reads | annotation | genes | product |
| \* | NC\_001604.1 | 35,095 | 0 | A | G | 98.8% | 4602.1 / NA | 1537 | intergenic (–/–) | *–/–* | –/– |
| Reads supporting (aligned to +/- strand):  **ref** base A (4/6);  **major** base G (712/795);  **minor** base T (13/6);  **total** (730/807) | | | | | | | | | | | |
| Fisher's exact test for biased strand distribution *p*-value = 1.03e-01 | | | | | | | | | | | |
| Kolmogorov-Smirnov test that lower quality scores support variant *p*-value = 9.99e-01 | | | | | | | | | | | |

Only 100 of 1537 total aligned reads displayed.  

`CTCTCTGACAAAGGCTTGGGGTCCAGCCGATGGCTACACGACCATCGAGTTACGTCGAGTAACCTCCACTACCGACCGATTGGTTGACTTTACGGATGGTTCAATCCTCCGCGCGTATGACCTTAACGTCGCTCAGATTCAAACGATGCACGTAGCGGAAGAGGCCCGTGACCTCACTACGGATACTATCGGTGTCAATAACGATGGTCACTTGGATGCTCGTGGTCGTCGAATTGTGAACCTAGCGAACGCCGTGGATGACCGCGATGCTGTTCCGTTTGGTCAACTAAAGACCATGAACCAGAACTCATGGCAAGCACGTAATGAAGCCTTACAGTTCCGTAATGAGGCTGAGACTTTCAGAAACCAAGCGGAGGGCTTTAAGAACGAGTCCAGTACCAACGCTACGAACACAAAGCAGTGGCGCGATGAGACCAAGGGTTTCCGAGACGAAGCCAAGCGGTTCAAGAATACGGCTGGTCAATACGCTACATCTGCTGGGAACTCTGCTTCCGCTGCGCATCAATCTGAGGTAAACGCTGAGAACTCTGCCACAGCATCCGCTAACTCTGCTCATTTGGCAGAACAGCAAGCAG  >  NC_001604.1/34797‑35392`  

                                                                                                                                                                                                                                                                                                          `|`                                                                                                                                                                                                                                                                                                           
 `tctctGACAAAGGCTTGGGGTCCAGCCGATGGCTACACGACCATCGAGTTACGTCGAGTAACCTCCACTACCGACCGATTGGTTGACTTTACGGATGGTTCAATCCTCCGCGCGTATGACCTTAACGTCGCTCAGATTCAAACGATGCACGTAGCGGAAGAGGCCCGTGACCTCACTACGGATACTATCGGTGTCAATAACGATGGTCACTTGGATGCTCGTGGTCGTCGAATTGTGAACCTAGCGAACGCCGTGGATGACCGCGATGCTGTTCCGTTTGGTCAACTAAAGACCATGGAcc                                                                                                                                                                                                                                                                                                        <  1:13760/301‑1 (MQ=255)`  
      `ggacagggCTTGGGGTCCAGCCGATGGCTACACGACCATCGAGTTACGTCGAGTAACCTCCACTACCGACCGATTGGTTGACTTTACGGATGGTTCAATCATCCGCGCGTATGACCTTAACGTCGCTCAGATTCAAACGATGCACGTAGCGGAAGAGGCCCGTGACCTCACTACGGATACTATCGGTGTCAATAACGATGGTCACTTGGATGCTCGTGGTCGTCGAATTGTGAACCTAGCGAACGCCGTGGATGACCGCGATGCTGTTCCGTTTGGTCAACTAAAGACCATGGACCAGAAc                                                                                                                                                                                                                                                                                                   <  1:159039/295‑1 (MQ=255)`  
      `gACAAAGGCTTGGGGTCCAGCCGATGGCGAAACGACCATCGAGTTACGTCGAGTAACCTCCACTACCGACCGATTGGTTGACTTTACGGATGGTTCAATCCTCCGCGCGTATGACCTTAACGTCGCTCAGATTCAAACGATGCACGTAGCGGAAGAGGCCCGTGACCTCACTACGGATACTATCGGTGTCAATAACGATGGTCACTTGGATGCTCGTGGTCGTCGAATTGTGAACCTAGCGAACGCCGTGGATGACCGCGATGCTGTTCCGTTTGGTCAACTAAAGACCATGGACCAGAAc                                                                                                                                                                                                                                                                                                   <  1:18401/301‑1 (MQ=255)`  
      `aaCAAAGGCGTGGGGTCCAGCCGATGGCTACACGACCATCGAGTTACGTCGAATAACCTCCACTACCGACCGATTGGTTGACTTTACGGATGGTTCAATCCTCCGCGCGTATGACCTTAACGTCGCTCAGATTCAAACGATGCACGTAGCGGAAGAGGCCCGTGACCTCACTACGGATACTATCGGTGTCAATAACAATGGTCACTTGGATGCTCGTGGTCGTCGAATTGTGAACCTAGCGAACGCCGTGGATGACCGCGATGCTGTTCCGTTTGGTCAACTAAAGACCATGGACCAGAAc                                                                                                                                                                                                                                                                                                   <  1:50717/300‑1 (MQ=255)`  
       `aCAAAGGCTTGGGGTCCAGCCGATGGCTACACGACCATCGAGTTACGTCGAGTAACCTCCACTACCGACCGATTGGTTGACTTTACGGATGGTTCAATCCTCCGCGCGTATGACCTTAACGTCGCTCAGATTCAAACGATGCACGTAGCGGAAGAGGCCCGTGACCTCACTACGGATACTATCGGTGTCAATAACGATGGTCACTTGGATGCTCGTGGTCGTCGAATTGTGAACCTAGCGAACGCCGTGGATGACCGCGATGCTGTTCCGTTTGGTCAACTAAAGACCATGGACCAGAACt                                                                                                                                                                                                                                                                                                  <  1:157104/301‑1 (MQ=255)`  
           `aGGCTTGGGGGCCAGCCGATGGCTACACGACCATCGAGTTACGTCGAGTAACCTCCACTACCGACCGATTGGTTGACTTTACGGATGGTTCAATCCTCCGCGCGTATGACCTTAACGTCGCTCAGATTCAAACGATGCACGTAGCGGAAGAGGCCCGTGACCTCACTACGGATACTATCGGTGTCAATAACGATGGTCACTTGGATGCTCGTGGTCGTCGAATTGTGAACCTAGCGAACGCCGTGGATGACCGCGATGCTGTTCCGTTTGGTCAACTAAAGACCATGGACCAGAACTCATg                                                                                                                                                                                                                                                                                              <  1:85438/301‑1 (MQ=255)`  
            `ggCTTTGGGTCCAGCCGATTGCTACACGACCATCGTGTTACGTCGAGTAACCTCCAATACCGGCCGATTGGTTGACTTTACTGATGGTTCAAGCCTCCGCGCGTATGACCTTAACGTCGCTCAGATTCAAACGATGCACGTAGCGGAAGAGGCCCGTGACCTCACTACGGATACTATCGGTGTCAATAACGATGGTCACTTGGATGCTCGTGGTCGTCGAATTGTGAACCTAGCGAACGCCGTGGATGACCGCGATGCTGTTCCGTTTGGTCAACTAAAGACCATGGACCAGAACTCATgg                                                                                                                                                                                                                                                                                             <  1:73383/301‑1 (MQ=255)`  
            `ggCTTGGGGTCCAGCCGATGGCTACACGACCATCGAGTTACGTCGAGTAACCTCCACTACCGACCGATTGGTTGACTTTACGGATGGTTCAATCCTCCGCGCGTATGACCTTAACGTCGCTCAGATTCAAACGATGCACGTAGCGGAAGAGGCCCGTGACCTCACTACGGATACTATCGGTGTCAATAACGATGGTCACTTGGATGCTCGTGGTCGTCGAATTGTGAACCTAGCGAACGCCGTGGATGACCGCGATGCTGTTCCGTTTGGTCAACTAAAGACCATGGACCAGAACTCATgg                                                                                                                                                                                                                                                                                             <  1:70691/301‑1 (MQ=255)`  
             `gCTTGGGGTCCAGCCGATGGCTACACGACCATCGAGTTACGTCGAGTAACCTCCACTACCGACCGATTGGTTGACTTTACGGATGGTTCAATCCTCCGCGCGTATGACCTTAACGTCGCTCAGATTCAAACGATGCACGTAGCGGAAGAGGCCCGTGACCTCACTACGGATACTATCGGTGTCAATAACGATGGTCACTTGGATGCTCGTGGTCGTCGAATTGTGAACCTAGCGAACGCCGTGGATGACCGCGATGCTGTTCCGTTTGGTCAACTAAAGACCATGGACCAGAACTCATGGc                                                                                                                                                                                                                                                                                            <  1:219293/301‑1 (MQ=255)`  
                   `ggTCCAGCCGATGGCTACACGACCATCGAGTTACGTCGAGTAACCTCCACTACCGACCGATTGGTTGACTTTACGGATGGTTCAATCCTCCGCGCGTATGACCTTAACGTCGCTCAGATTCAAACGATGCACGTAGCGGAAGAGGCCCGTGACCTCACTACGGATACTATCGGTGTCAATAACGATGGTCACTTGGATGCTCGTGGTCGTCGAATTGTGAACCTAGCGAACGCCGTGGATTACCGCGATGCTGTTCCGTTTGGTCAACTAAAGACCATGGACCAGAACTCATGGCAAGCAc                                                                                                                                                                                                                                                                                      >  1:223660/1‑301 (MQ=255)`  
                         `gCCGAGGGCTACACGACCAACGAGTTACGTCGAGTAACCTCCACTGCCGAACGATTGGTTGACTTTACGGATGGTTCAATCCTCCGCGCGTATGACCTTAACGTCGCTCAGATTCAAACGATGCACGTAGCGGAAGAGGCCCGTGACCTCACTACGGATACTATCGGTGTCAATAACGATGGTCACTTGGATGCTCGTGGTCGTCGAATTGTGAACCTAGCGAACGCCGTGGATGACCGCGATGCTGTTCCGTTTGGTCAACTAAAGACCATGGACCAGAACTCATGGCAAGCACGTAATg                                                                                                                                                                                                                                                                                <  1:94240/301‑1 (MQ=255)`  
                              `tGGCTACACGACCATCGAGTTACGTCGAGTAACCTCCACTACCGACCGATTGGTTGACTTTACGGATGGTTCAATCCTCCGCGCGTATGACCTTAACGTCGCTCAGATTCAAACGATGCACGTAGCGGAAGAGGCCCGTGACCTCACTACGGATACTATCGGTGTCAATAACGATGGTCACTTGGATGCTCGTGGTCGTCGAATTGTGAACCTAGCGAACGCCGTGGATGACCGCGATGCTGTTCCGTTTGGTCAACTAAAGACCATGGAACAGAACTCATGGCAAGCACGTAATGAAGcc                                                                                                                                                                                                                                                                           >  1:192802/1‑301 (MQ=255)`  
                                    `taagagacaggAGTTACGTCGAGTAACCTCCACTATCGACCGATTGGTTGACTTTACGGATGGTTCAATCCTCCGCGCGTATGACCTTAACGTCGCTCAGATTCAAACGATGCACGTAGCGGAAGAGGCCCGTGACCTCACTACGGATACTATCGGTGTCAATAACGATGGTCACTTGGATGCTCGTGGTCGTCGAATTGTGAACCTAGCGAACGCCGTGGATGACCGCGATGCTGTTCCGTTTGGTCAACTAAAGACCATGGACCAGAACTCATGGCAAGTACGTAATGAAGCCTTACAg                                                                                                                                                                                                                                                                     <  1:82854/291‑1 (MQ=255)`  
                                     `agaaCCAGCGAGTAACGGCGAGTAACCTCCACTACCGACCGATTGGTTGAATTTACGGATGGTTCAATCATCCGCGCGTATGACCTTAACGTCGCTCAGATTCAAACGATGAACGTAGCGGAAGAGGCACGTGACCTCACTACGGATACTATCGGTGTCAATAACGATGGTCACTTGGATGCTCGTGGGCGTCGAATTGTGAACCTAGCGAACGCCGTGGATGACCGCGATGCTGTTCCGTTTGGTCAACTAAAGACCATGGACCAGAACTCATGGCAAGCACGTAATGAAGCCTTACAGt                                                                                                                                                                                                                                                                    <  1:313935/298‑1 (MQ=255)`  
                                           `aTCGAGTTACGTCGAGTAACCTCCACTACCGACCGATTGGTTGACTTTACGGATGGTTCAATCCTCCGCGCGTATGACCTTAACGTCGCTCAGATTCAAACGATGCACGTAGCGGAAGAGGCCCGTGACCTCACTACGGATACTATCGGTGTCAATAACGATAGTCACTTGGATGCTCGTGGTCGTCGAATTGTGAACCTAGCGAACGCCGTGGATGACCGCGATGCTGGTCCGTTTGGTCAACTAAAGACCATGGACCAGAACTCATGGCAAGCACGTAATGAAGCCTTACAGTTCCGTa                                                                                                                                                                                                                                                              >  1:179489/1‑301 (MQ=255)`  
                                            `tCGAGTTACGTCGAGTAACCTCCACTACCGACCGATTGGTTGACTTTACGGATGGTTCAATCCTCCGCGCGTATGACCTTAACGTCGCTCAGATTCAAACGATGCACGTAGCGGAAGAGGCCCGTGACCTCACTACGGATACTATCGGTGTCAATAACGATGGTCACTTGGATGCTCGTGGTCGTCGAATTGTGAACCTAGCGAACGCCGTGGATGACCGCGATGCTGTTCCGTTTGGTCAACTAAAGACCATGGACCAGAACTCATGGCAAGCACGTAATGAAGCCTTACAGTTACGTaa                                                                                                                                                                                                                                                             <  1:190787/301‑1 (MQ=255)`  
                                            `ggaaatgtgtataaaggccaggcACTGCTGACCGATTGGTTGACTTAACAGATGGTTCAATCCGCCGAGCGTATAACCATAACGACGATCAGATTCAAACTATGCACGTAGCGGAAGAGGCCCATGACCTCACTACGGATACAATCGGTGTCAATAGCGATGGTCACTTGGATGTTCGTGGTCGTCGGATTGTGAACCTAGCGAACGCCGTGGATGACCGCGATGCTGTTCCGTATGGTCAACTAAAGGCCATGGACCAGAACTCATGGCAAGCACGTAATGAAGCCTTACAGTTCCGTaa                                                                                                                                                                                                                                                             <  1:330172/279‑1 (MQ=255)`  
                                                        `gAGTAACCTCCACTACCGACCGATTGGTTGACTTTACGGATGGTTCAATCCTCCGCGCGTATGACCTTAACGTCGCTCAGATTCAAACGATGCACGTAGCGGAAGAGGCCCGTGACCTCACTACGGATACTATCGGTGTCAATAACGATGGTCACTTGGATGCTCGTGGTCGTCGAATTGTGAACCTAGCGAACGCCGTGGATGACCGCGATGCTGTTCCGTTTGGTCAACTAAAGACCATGGACCAGAACTCATGGCAAGCACGTAATGAAGCCTTACAGTTCCGTAATGAGGCTGAGAc                                                                                                                                                                                                                                                 <  1:112020/301‑1 (MQ=255)`  
                                                              `ccTCCACTACCGACCGATTGGTTGACTTTACGGATGGTTCAATCCTCCGCGCGTATGACCTTAACGTCGCTCAGATTCAAACGATGCACGTAGCGGAAGAGGCCAGTGACCTCACTACGGATACTATCGGTGTCAATAACGATGGTCACTTGGATGCTCGTGGTCGTCGACTTGTGAACCTAGCGAACGCCGTGGATGACCGCGATGCTGTTCCGTTTGGTCAACTAAAGACCATGGACCAGCACTCATGGCCAGCACGTCATGAAGCCTTACAGTTCCGtctgttccttatacacatctc                                                                                                                                                                                                                                           >  1:317012/1‑281 (MQ=255)`  
                                                               `cTCCACTACCGACCGATTGGTTGACTTTACGGATGGTTCAATCCTCCGCGCGTATGACCTTAACGTCGCTCAGATTCAAACGATGCACGTAGCGGAAGAGGCCCGTGACCTCACTACGGATACTATCGGTGTCAATAACGATGGTCACTTGGATGCTCGTGGTCGTCGAATTGTGAACCTAGCGAACGCCGTGGATGACCGCGATGCTGTTCCGTTTGGTCAACTAAAGACCATGGACCAGAACTCATGGCAAGCACGTAATGAAGCCTTAAAGTTCCGTAATGAGGCTGAGACTTTCAGa                                                                                                                                                                                                                                          >  1:104066/1‑301 (MQ=255)`  
                                                                          `accgaTTGGTTGACTTTACGGATGGTTCAATCCTCCGGGCGTATGACCTTAACGGCGCTCAGATTCAAACGATGCACGTAGCGGAAGAGGCCCGTGACCTCACTACGGATACTATCGGTGTCAATAACGATGGTCACTTGGATGCCCGTGGTCGTCGAATTGTGAACCTAGCTAACGCCGTGGATGACCGCGATGCTGTTCCGTTTGGTCAACTAAAGACCATGGACCAGAACTCATGGCAAGCACGTAATGAAGCCTTACAGTTCCGTAATGAGGCTGAGACTTTCAGAAACCAAGCGGa                                                                                                                                                                                                                               <  1:77836/301‑1 (MQ=255)`  
                                                                           `ccgaTTGGTTGACTTTACGGATGGTTCAATCCTCCGGGCGTATGACCTTAACGTCGCTCAGATTCAAACGATGCACGTATCGGAAGAGGCCCGTGACCTCACTACGGATACTATCGGTGTCAATAACGATGGTCACTTGGATGCTCGTGGTCGTCGAATTGTGAACCTAGCGAACGCCGTGGATGACCGCGATGCTGTTCCGTTTGGTCAACTAAAGACCATGGACCAGAACTCATGGCAAGCACGTAATGAAGCCTTACAGTTCCGTAATGAGGCTGAGACTTTCAGAAACCAAGCGGAg                                                                                                                                                                                                                              <  1:48240/301‑1 (MQ=255)`  
                                                                                    `tGACTTTACGGATGGTTCAATCCTCCGCGCGTATGACCTTAACGTCGCTCAGATTCAAACGATGCACGTAGCGGAAGAGGCCCGTGACCTCACTACGGATACTATCGGTGTCAATAACGATGGTCACTTGGATGCTCGTGGTCGTCGAATTGTGAACCTAGCGAACGCCGTGGATGACCGCGATGCTGTTCCGTTTGGTCAACTAAAGACCATGGACCAGAACTCATGGCAAGCACGTAATGAAGCCTTACAGTTCCGTAATGAGGCTGAGACTTTCAGAAACCAAGCGGAGGGCTTTAAg                                                                                                                                                                                                                     <  1:5801/301‑1 (MQ=255)`  
                                                                                      `tataagagacaggTTCAATCCTCCGCGCGTATGACCTTAACGTCGCTCAGATTCAAACGATGCACGTAGCGGAAGAGGCCCGTGACCTCACTACGGATACTATCGGTGTCAATAACGATGGTCACTTGGATGCTCGTGGTCGTCGAATTGTGAACCTAGCGAACGCCGTGGATGACCGCGATGCTGTTCCGTTTGGTCAACTAAAGACCATGGACCAGAACTCATGGCAAGCACGTAATGAAGCCTTACAGTTCCGTAATGAGGCTGAGACTTTCAGAAACCAAGCAGAGGGCTTTAAGaa                                                                                                                                                                                                                   <  1:320586/290‑1 (MQ=255)`  
                                                                                      `aCTTTACGGATGGTTCAATCCTCCGCGCGTATGACCTTAACGTCGCTCAGATTCAAACGATGCACGTAGCGGAAGAGGCCCGTGACCTCACTACGGATACTATCGGTGTCAATAACGATGGTCACTTGGATGCTCGTGGTCGTCGAATTGTGAACCTAGCGAACGCCGTGGATGACCGCGATGCTGTTCCGTTTGGTCAACTAAAGACCATGGACCAGAACTCATGGCAAGTACGTAATGAAGCCTTACAGTTCCGTAATGAGGCTAAGACTTTCAGAAACCAAGCGGAGGGCTTTAAGaa                                                                                                                                                                                                                   >  1:261017/1‑301 (MQ=255)`  
                                                                                      `aCTTTACGGATCGTTCAATCCTCCGCGGGTATGACCTTAACGTCGCTCAGATTCAATCGATGCACGTAGCGGAAGAGGCCCGTGACCTCACTACGGATACTATCGGTGTCAATAACGATGGTCACTTGGATGCTCGTGGTCGTCGAATTGTGAACCTAGCGAACGCCGTGGATGACCGCGATGCTGTTCCGTTTGGTCAACTAAAGACCATGGACCAGAACTCATGGCAAGCACGTAATGAAGCCTTACAGTTCCGTAATGAGGCTGAGACTTTCAGAAACCAAGCGGAGGGCTTTAAGaa                                                                                                                                                                                                                   <  1:305788/301‑1 (MQ=255)`  
                                                                                       `cTTTATGGATGGTTCAATCCTCCGCGCGTATGACCTTAACGTCGCTCAGATTCAAACGATGCACGTAGCGGAAGAGGCCCGTGACCTCACTACGGATACTATCGGTGTCAATAACGATGGTCACTTGGATGCTCGTGGTCGTCGAATTGTGAACCTAGCGAACGCCGTGGATGACCGCGATGCTGCTCCGTTTGGTCAACTAAAGACCATGGACCAGAACTCATGGCAAGCACGTAATGAAGCCTTACAGTTCCGTAATGAGGCTGAGACTTTCAGAAACCAAGCGGAGGGCTTTAAGaaa                                                                                                                                                                                                                  >  1:80390/1‑300 (MQ=255)`  
                                                                                            `cGGATGGTTCAATCCTCCGCGCGTATGACCTTAACGTCGCTCAGATTCAAACGATGCACGTAGCGGAAGAGGCCCGTGACCTCACTACGGATACTATCGGTGTCAATAACGATGGTCACTTGGATGCTCGTGGTCGTCAAATTGTGAACCTAGCGAACGCCGTGGATGACCGCGATGCTGTTCCGTTTGGTCAACTAAAGACCATGGACCAGAACTCATGGCAAGCACGTAATGAAGCCTTACAGTTCCGTAATGAGGCTGAGACTTTCAGAAACCAAGCGGAGGGCTTTAAGAACGAGTc                                                                                                                                                                                                             <  1:186272/301‑1 (MQ=255)`  
                                                                                                  `gTTCAATCCTCCGCGCGTATGACCTTAACGTCGCTCAGATTCAAACGATGCACGTAGCGGAAGAGGCCCGTGACCTCACTACGGATACTATCGGTGTCAATAACGATGGTCACTTGGATGCTCGTGGTCGTCGAATTGTGAACCTAGCGAACGCCGTGGATGACCGCGATGCTGTTCCGTTTGGTCAACTAAAGACCATGGACCAGAACTCATGGCAAGCACGTAATGAAGCCTTACAGTTCCGTAATGAGGCTGAGACTTTCAGAAACCAAGCGGAGGGCTTTAAGAACGAGTCCAGTAc                                                                                                                                                                                                       >  1:14808/1‑301 (MQ=255)`  
                                                                                                  `gTTCAATCCTCCGCGCGTATGACATTAACGTCGCTCAGATTCAAACGATGCACGTAGCAGAAGAGGCCCGTGACCTCACTACGGATACTATCGGTGTCAATAACGATGGTCACTTGGATGCTCGTGGTCGTCGAATTGTGAACCTAGAGAACGCCGTGGATGACCGCGATGCTGTTCCGTTTGGTCAACTAAAGACCATGGACCAGAACTCATGGCAAGCACGTACTGAAGCCTTACAGTTCCTTAATGAGGCTGAGACTTTCAGAAACCAAGCGGAGGGcctgtctcttatacacatctc                                                                                                                                                                                                       >  1:2022/1‑281 (MQ=255)`  
                                                                                                  `gTTCAATCCTCCGCGCGTACGACCTTAACGTCGCTCAGATTCAAACGATGCACGTAGCGGAAGAGGCCCGTGACCTCACTACGGATACTATCGGTGTCAATAACGATGGTCACTTGGATGCTCGTGGTCGTCGAATTGTGAACCTAGCGAACGCCGTGGATGACCGCGATGCTGTTCCGTTTGGTCAACTAACGACCATTGACCAGAACTCATGGCAAGCACGTAATGAAGCCTTACAGTTACGTACTGAGGCTGAGAATTTCAGAAACCAAGCGGAGGGcctgtctcttatacaaatatc                                                                                                                                                                                                       >  1:302521/1‑281 (MQ=255)`  
                                                                                                        `tactccGCGCGTATGACCTTAACTTCGCTCAGATTCAAACGATGCACGTAGCGGAAGAGGCCCGTGACCTAACTACGGATACTAGCGGTGTCAATAACGATGGTCACTTGGATGCTCGGGGTCTTCGAATTGTGAACCTAGCGAACGCCGTGGATGACCGCGATGCTGTTCCGTTTGGTCAACTAAAGACCATGGACCAGAACTCATGGAAAGCACGTAATGAAGCCTTACAGTTCCGTAATGAGGCTGATACTTTCAGAAACCAAGCGGAGGGCTTTAAGAACGAGTCCAGTACCAACGc                                                                                                                                                                                                 <  1:162329/299‑1 (MQ=255)`  
                                                                                                          `ctccGCGCGTATGACCTTAACGTCGCTCAGATTCAAACGATGCACGTAGCGGAAGAGGACCGTGACCTCACTACGGATACTATCGGTGTCAATAACGATGGTAACTTGGATGCTCGTGGTCGTCGAATTGTGAACCTAGCGAACGCCGTGGATGACCGCGATGCTGTTCCGTTTGGTCAACTAAAGACCATGGACCAGAACTCATGGCAAGCACGTAATGAAGCCTTACAGTTCCGTAATGAGGCTGAGACTTTCAGAAACCAAGCGGAGGGCTTTAAGAACGAGTCCAGTACAAACGCTa                                                                                                                                                                                               >  1:44610/1‑301 (MQ=255)`  
                                                                                                                 `cgTATGACCTTAACGTCGCTCAGATTCAAACGATGCACGTAGCGGAAGAGGCCCGTGACCTAACTACGGATACTATCGGTGTCAATAACGATGGTCACTTGGATGCTCGTGGTCGTCGAATTGTGAACCTAGCGAACGCCGTGGATGACAGCGATGCTGTTCCGTTTGGTCAACTAAAGACCATGGACCAGAACTCATGGCAAGCACGTAATGAAGCCTTACAGTTCCGTAATGAGGCTGAGACTTTCAGAAACCAAGCGGAGGGCTTTAAGAACGAGTACAGTACCAACGCTACGAacaa                                                                                                                                                                                        >  1:36438/1‑300 (MQ=255)`  
                                                                                                                       `aCCTTAACGTCGCTCAGATTCAAACGATGCACGTAGCGGAAGAGGCCCGTGACCTCACTACGGATACTATCGGTGTCAATAACGATGGTCACTTGGATGCTCGTGGTCGTCGAATTGTGAACCTAGCGAACGCCGTGGATGACCGCGATGCTGTTCCGTTTGGTCAACTAAAGACCATGGACCAGAACTCATGGCAAGCACGTAATGAAGCCTTACAGTTCCGTAATGAGGCTGAGACTTTCAGAAACCAAGCGGAGGGCTTTAAGAACGAGTCCAGTACCAACGCTACGAACACAAAgaa                                                                                                                                                                                  >  1:63054/1‑299 (MQ=255)`  
                                                                                                                       `aCCTTAACATCGCTCAGATTCAAACTATGCACGTAGCGGAAGAGGCCCGTGACCTCACTACGGATACTATCGGTGTCAATAACGATGGTCACTTGGATGCTCGTGGTCGTCGAATTGTGAACCTAGCGAACGCCGTGGATGACCGCGATGCTGTTCCGTTTGGTCAACTAAAGACCATGGACCAGAACTCATGGCAAGCACGTAATGAAGCCTTACAGTTCCGTAATGATGCTGAGACTTTCAGAAACCAAGCGGAGGGCTTTAAGAACGATTCCAGTACCAACGCTACGAACACAAAGCa                                                                                                                                                                                  >  1:337666/1‑301 (MQ=255)`  
                                                                                                                        `ccTTAACGTCGCTCAGATTCAAACGATGCACGTAGCGGAAGAGGCCCGTGACCTCACTACGGATACTATCGGTGTCAATAACGATGGTCACTTGGATGCTCGTGGTCGTCGAATTGTGAACCTAGCGAACGCCGTGGATGACCGCGATGCTGTTCCGTTTGGTCAACTAAAGACCATGGACCAGAACTCATGGCAAGCACGTAATGAAGCCTTACAGTTCCGTAATGAGGCTGAGACTTTCAGAAACCAAGCGGAGGGCTTTAAGAACGATTCCAGTACCAACGCTACGAACACaaactgt                                                                                                                                                                                 >  1:300457/1‑297 (MQ=255)`  
                                                                                                                        `ccTTAACGTCGCTCAGATTCAAACGATGCACGTAGCGGAAGAGGCCCGTGACCTCACTACGGATACTATCGGTGTCAATAACGATGGTCACTTGGATGCTCGTGGTCGTCGAATTGTGAACCTAGCGAACGCCGTGGATGACCGCGATGCTGTTCCGTTTGGTCAACTAAAGACCATGGACCAGAACTCATGGCAAGCACGTAATGAAGCCTTACAGTTCCGTAATGAGGCTGAGACTTTCAGAAACCAAGCGGAGGGCTTTAAGAACGAGTCCAGTACCAACGCTACGAACACCAAGccc                                                                                                                                                                                 >  1:9732/1‑299 (MQ=255)`  
                                                                                                                         `cggagatgtgtataagagacagcGATGCACGTAGCTGAAGAGGCCCGTGACCTCACTACGGATACTATCGGTGTCAATAACGATGGTCACTTGGATGCTCGTGGTCGTCGAATTGTGAACCTAGCGAACGCCGTGGATGACCGCGATGCTGTTCCGTTTGGTCAACTAAAGACCATGGACCAGAACTCATGGCAAGCACGTAATGAAGCCTTACAGTTCCGTAATGAGGCTGAGACTTTCAGAAACCAAGCGGAGGGCTTTAAGAACGAGTCCAGTACCAACGCTACGAACACAAAGCAGt                                                                                                                                                                                <  1:245223/279‑1 (MQ=255)`  
                                                                                                                                  `gacaggATTCAAACGATGCACGTAGCGGAAGAGGCCCGTGACCTCACTACGGATACTATCGGTGTCAATAACGATGGTCACTTGGATGCTCGTGGTCGTCGAATTGTGAACCTAGCGAACGCCGTGGATGACCGCGATGCTGTTCCGTTTGGTCAACTAAAGACCATGGACCAGAACTCATGGCAAGCACGTAATGAAGCCTTACAGTTCCGTAATGAGGCTGAGACTTTCAGAAACCAAGCGGAGGGCTTTAAGAACGAGTCCAGTACCAACGCTACGAACACAAAGCAGTGGCGCGATg                                                                                                                                                                       <  1:115273/296‑1 (MQ=255)`  
                                                                                                                                              `actaggCAGGTAGAGGAAGAGGCCCGTGACCTCACTACGGATACTATCGGTGTCAATAACGATGGTCACTTGGATGATCGTGGTCGTCGAATTGTGAACCTAGCGAACGCCGTGGATGACCGCGATGCTGTTCCGTTTGGTCAACTAAAGACCATGGACCAGAACTCATGGCAAGCACGTAATGAAGCCTTACAGTT‑CGTAATGAGGCTGAGACTTTCAGAAACCAAGCGGAGGGCTTTAAGAACGAGTCCAGTACCAACGCTACGAACACAAAGCAGTGGCGCGATGAGACCAAGGGtag                                                                                                                                                          <  1:134293/296‑3 (MQ=255)`  
                                                                                                                                                 `aTGCACGTAGCGGAAGAGGCACGTGACCTCACTACGGATACTATCGGTGTCAATAACGATGGTCACTTGGATGCTCGTGGTCGTCGAATTGTGAACCTAGCGAACGCCGTGGATGACCGCGATGCTGTTCCGTTTGGTCAACTAAAGACCATGGACCAGAACTCATGGCAAGCACGTAATGAAGCCTTACAGTTCCGTAATGAGGCTGAGACTTTCAGAAACCAAGCGGAGGGCTTTAAGAACGAGTCCAGTACCAACGCCACGAACACAAAGCAGTGgcgctgtctcttatacacatctc                                                                                                                                                        >  1:206833/1‑282 (MQ=255)`  
                                                                                                                                                      `cgtcgtggAAGAGGCCCGTGACCTCACTACGGATACTATCGGTGTCAATAACGATGGTCACTTGGATGCTCGTGGTCGTCGAATTGTGAACCTAGCGAACGCCGTGGATGACCGCGATGCTGTTCCGTTTGGTCAACTAAAGACCATGGACCAGAACTCATGGCAAGCACGTAATGAAGCCTTACAGTTCCGTAATGAGGCTGAGACTTTCAGAAACCAAGCGGAGGGCTTTAAGAACGAGTCCAGTACCAACGCTACGAACACAAAGCAGTGGCGCGATGAGACCAAGGGTTTCCGAGAc                                                                                                                                                   >  1:127028/7‑301 (MQ=255)`  
                                                                                                                                                      `cGTAGTGGCCGAGGCCCGTGACCTCACTACGGATACTATCGGTGTCAATAACGATGGTCACTTGGATGCTCGTGGTCGTCGAATTGTGAACCTAGCGAACGCCGTGGATGACCGCGATGCTGTTCCGTTTGGTCAACTAAAGACCATGGACCAGAACTCATGGCAAGCACGTACTGAAGCCTTACAGTTCCGTAATGAGGCTGAGACTTTCAGAAAACTAGCGAAGGGCTTTAAGAACGAGTCCAGTACCAACGCTACGAACCCAAAGCAGTGGCGCGATGAGACCAAGGGTTTCCGAGAc                                                                                                                                                   >  1:174620/1‑301 (MQ=255)`  
                                                                                                                                                      `cGTAGCCGAAGCGGCCCGTCACCTCACTGCGGATACTATCGGTGCCTATAACGTTTGTCACTTGGATGCTCGTTGTCGTCGAATTGTTAACCTAGCGAACTGCGTGGTTCACCGGGATGCTGTTCCGTTTTGTCAACTAACGTCCATTGACCAGAACTCATGGCAAACACGTAATGAAGCCTTACAGTTCCGTAATGAAGCTGAGACTTTCAGAAACCAAGCGGAGGGCTTTAAGAACGAGTCCAGTACCAACGCTACGAACACAAAGCAGTGGCGCGATGAGACCAAGGGTTTCCAAGAc                                                                                                                                                   <  1:290555/301‑1 (MQ=255)`  
                                                                                                                                                        `gaGCGGACGAGCCCCGTCACCTGACTACGGATACTATCGGTGGCAATAACGTTAGTTACTTGGATGCTCGTGGTCGGCGAATTGTGAACCAAGCGAAGGCCTGGGATGACCGCGATGCTGCTCCGTTTGGGCAACTAAAGATCATGGACCATAACTCATGGCAAGCACGTAATGAAGCCTTACAGTTCCGTAATGAGGCTGAGACTTTCAGAAACCAAGCGGAGGGCTTTATGAACGAGTCCAGTACCAACGCTACGAACACAAAGCAGTGGCGCGATGAGACCAAGGGTTTCCGAGACGa                                                                                                                                                 <  1:106836/300‑1 (MQ=255)`  
                                                                                                                                                           `gggAAGAGGCCCGTGTCCTGACTACGGATACGAGCGGTGTCAATAACGATGGTCAATTGGATGCTCGTAGTAGTCTAATTGTGAACCTATCAAACGCCGTGGATGACCGCGATGCTGTTCCGTTTGGTCAACTAAAGACCATGGACCAGAACTCAAGGCAAGCACGTAATGAAGCGTTACGGTTCCGTAATGAGGCTGAGACTATCAGAAACCGAGCGGAGGGCTTTAAGGACGAGTCCAGTACCAACGCTACGAACACGAAGCAGTGGCGCGATGAGACCAAGGGTTTCCGAGACGAAGc                                                                                                                                              <  1:195347/300‑1 (MQ=255)`  
                                                                                                                                                                    `cccGTGACCTCACTACGGATACTATCGGTGTCAATAACGATGGTCACTTGGATGCTCGTGGTCGTCGAATTGTGAACCTAGCGAACGCCGTGGATGACCGCGATGCTGTTCCGTTTGGTCAACTAAAGACCATGGACCAGAACTCATGGCAAGCACGTAATGAAGCCTTACAGTTCCGTAATGAGGCTGAGACTTTCAGAATCCAAGCGGAGGGCTTTAAGAACGAGTCCAGTACCAACGCTACGAACACAAAGCAGTGGCGCGATGAGACCAAGGGTTTCCGAGACGAAGCCAAGCGGtt                                                                                                                                     >  1:67503/1‑301 (MQ=255)`  
                                                                                                                                                                          `gtgtataagagacagcTATCGGTGTCAATAACGATGGTCACTTGGATGCTCGTGGTCGTCGAATTGTGAACCTAGCGAACGCCGTGGATGACCGCGATGCTGTTCCGTTTGGTCAACTAAAGACCATGGACCAGAACTCATGGCAAGCACGTAATGAAGCATTACAGTTCCGTAATGAGGCTGAGACTTTCAGAAACCAAGCGGGGGGCTTTAAGAACGAGTCCAGTACCAACGCTACGAACACAAAGCAGTGGCGCGATGAGACCAAGGGTTTCCGAGACGAAGCCAAGCGGTTCAAGaa                                                                                                                               <  1:61175/286‑1 (MQ=255)`  
                                                                                                                                                                          `aCCTCACTACGGATACTATCGGTGTCAATAACGATGGTCACTTGGATGCTCGTGGTCGTCGAATTGTGAACCTAGCGAACGCCGTGGATGACCGCGATGCTGTTCCGTTTGGTCAACCAAAGACCATGGACAAGAACTCATGGCAAGCACGTAATGAAGCCTTACAGTTCCGTAATGAGGCTGAGACTTTCAGAAACCAAGCGGAGGGCTTTAAGAACGAGTCCAGTACCAACGCTACGAACACAAAGCAGTGGCGCGATGAGACCAAGGGTTTCCGAGACGAAGCCAAGCGGTTCAAGaa                                                                                                                               <  1:30872/301‑1 (MQ=255)`  
                                                                                                                                                                          `aCCTCACTACGGATACTATCGGTGTCAATAACGATGGTCACTTGGATGCTCGTGGCCGTCGAATTGTGAACCTAGCGAACGCCGTGGATGACCGCGATGCTGTTCCGTTTGGTCAACTAAAGACCATGGACCAGAACTCATGGCAAGCACGTAATGAAGCCTTACAGTTCCGTAATGAGGCTGAGACTTTCAGAAACCAAGCGGAGGGCTTTAAGAACGAGTCCAGTACCAACGCTACGAACACAAAGCAGTGGCGCGATGAGACCAAGGGTTTCCGAGACGAAGCCGAGCGGTTCAAGaa                                                                                                                               <  1:332381/301‑1 (MQ=255)`  
                                                                                                                                                                          `aCCTAACTACGGATACTATCGGTTGAAATAACGATGGTCACTTGGATGCTCGTGGTCGTCGAATTGTGAACCTAGCGAACGCCGTGGATGACCGCGATGCTGTTCCGTTTGGTCAACTAAAGACCATGGACCAGAACTCATGGCAAGCACGTAATGAAGCCTTACAGTTCCGTAATGAGGCTGAGACTTTCAGAAACCAAGCGGAGGGCTTTAAGAACGAGTCCAGTACCAACGCTACGAACACAAAGCAGTGGCGCGATGAGACCAAGGGTTTCCGAGACGAAGCCGAGCGGTTCAAGaa                                                                                                                               <  1:296833/301‑1 (MQ=255)`  
                                                                                                                                                                              `cACTACGGATACTATCGGTGTCAATAACGATGGTCACTTGGATGCTCGTGGTCGTCGAATTGTGAACCTAGCGAACGCCGTGGATGACCGCGATGCTGTTCCGTTTGGTCAACTAAAGACCATGGACCAGAACTCATGGCAAGCACGTAATGAAGCCTTACAGTTCCGTAATGAGGCTGAGACTTTCAGAAACCAAGCGGAGGGCTTTAAGAACGAGTCCAGTACCAACGCTACGAACACAAAGCAGTGGCGCGATGAGACCAAGGGTTTCCGAGACGAAGCCGAGCGGTTCAAGAATACg                                                                                                                           >  1:278461/1‑301 (MQ=255)`  
                                                                                                                                                                              `cACTACGGATACTATCGGTGTCAATAACGATGGTCACTTGGATGCTCGTGGTCGTCGAATTGTGAACCTAGCGAACGCCGTGGATGACCGCGATGCTGTTCCGTTTGGTCAACTAAAGACCATGGACCAGAACTCATGGCAAGCACGTAATGAAGCCTTACAGTTCCGTAATGAGGCTGAGACTTTCAGAAACCAAGCGGAGGGATTTAAGAACGAGTCCAGTACCAACGCTACGAACACAAAGCAGTGGCGCGATGAGACCAAGGGTTTCCGAGACGAAGCCGAGCGGTTCAAGAATACg                                                                                                                           >  1:88210/1‑301 (MQ=255)`  
                                                                                                                                                                                `cTACGGATACTATCGGTGTCAATAACGATGGTCACTTGGATGCTCGTGGTCGTCGAATTGTGAACCTAGCGAACGCCGTGGATGACCGCGATGCTGTTCCGTTTGGTCAACTAAAGACCATGGACCAGAACTCATGGCAAGCACGTAATGAAGCCTTACAGTTCCGTAATGAGGCTGAGACTTTCAGAAACCAAGCGGCGGGCTTTAAGAACGAGTCAAGTACCAAAGCTACGAACACAAAGCAGTTGCGCGATGAGACCAAGGGTTTCCGAGACGAAGCCAACCGGTTCAAGAATACGGc                                                                                                                         >  1:293296/1‑301 (MQ=255)`  
                                                                                                                                                                                `cTACGGATACTATCGGTGTCAATAACGATGGTCACTTGGATGCTCGTGGTCGTCGAATTGTGAACCTAGCGAACGCCGTGGATGACCGCGATGCTGTTCCGTTTGGTCAACTAAAGACCATGGACCAGAACTCATGGCAAGCACGTAATGAAGCCTTACAGTTCCGTAATGAGGCTGAGACTTTCAGAAACCAAGCGGAGGGCTTTAAGAACGAGTACAGTACCAACGCTACGAACACAAAGCAGTGGCGCGATGAGACCAAGGGTTTCCGAGACGAAGCCGAGCGGTTCAAGAATACGGc                                                                                                                         <  1:123980/301‑1 (MQ=255)`  
                                                                                                                                                                                `atACGGATACTATCGGTGTCAATAACGATGGTCACTTGGATGCTCGTGGTCGTCGAATTGTGAACCTAGCGAACGCCGTGGATGACCGCGATGCTGTTCCGTTTGGTCAACTAAAGACCATGGACCAGAACTCATGGCAAGCACGTAATGAAGCCTTACAGTTCCGTAATGAGGCTGAGACTTTCAGAAACCAAGCGGAGGGCTTTAAGAACGAGTCCAGTACCAACGCTACGAACACAAAGCAGTGGCGCGATGAGAACAAGGGTTCCCGAGACGAAGCCGAGCGGCTCAAGaactgtct                                                                                                                         >  1:102425/2‑295 (MQ=255)`  
                                                                                                                                                                                   `agacagcTATCGGTGTCAATAACGATGGTCACTTGGATGCTCGTGGTCGTCGAATTGTGAACCTAGCGAACGCCGTGGATGACCGCGATGCTGTTCCGTTTGGCCAACTAAAGACCATGGACCAGAACTCATGGCAAGCACGTAATGAAGCCTTACAGTTCCGTAATGAGGCTGAGACTTTCAGAAACCAAGCGGAGGGCTTTAAGAACGAGTCCAGTACCAACGCTACGAACACAAAGCAGTGGCGCGATGAGACCAAGGGTTTCCGAGACGAAGCCGAGCGGTTCAAGAATACGGCTgg                                                                                                                      <  1:227335/295‑1 (MQ=255)`  
                                                                                                                                                                                    `ggATACTATCGGTGTCAATAACGATTGTCACTTCGATGCTCGTGGTCGTCGAATTGTGAACCTAGCGAACGCCGTGGATGACCGCGATGCTGTTCCGTTTGGTAAACTAAAGACCATGGACCAGAACTCATGGCAAGCACGTAATGAAGCCTTACAGTTCCGTAATGAGGCTGAGACTTTCAGAAACCAAGCGGAGGGCTTTAAGAACGAGTCCAGTACCAACGCTACGAACACAAAGCAGTGGCGCGATGAGACCAAGGGTTTCCGAGACGAAGCCGAGCGGTTCAAGAATACGGCTggg                                                                                                                     >  1:41372/1‑300 (MQ=255)`  
                                                                                                                                                                                    `ggATACTATCGGTGTCAATAACGATGGTCACTTGGATGCTCGTGGTCGTCGAATTGTGAACCTAGCGAACGCCGTGGATGACCGCGATGCTGTTCCGTTTGGTCAACTAAAGAACATGGACCAGAACTCATGGCAAGCACGTAATGAAGCCTTACAGTTCAGTAATGAGGCTGAGACTTTCAGAAACCACGCGGAGGGCATTAAGAACGAGTCTAGTACCAACGACACGAACACAAATCAGTGGCGCGATGTGACCAAGGGGTTTCGAGACGAAGCGGCGCGGATCAAGAATACGGCTGGt                                                                                                                     >  1:23117/1‑301 (MQ=255)`  
                                                                                                                                                                                     `gATACTATCGGTGTCAATAACGATGGTCACTTGGATGCTCGTGGTCGTCGAATTGTGAACCTAGCGAACGCCGTGGATGACCGCGATGCTGTTCCGTTTGGTCAACTAAAGACCATGGACCAGAACTCATGGCAAGCACGTAATGAAGCCTTACAGTTCCGTAATGAGGCTGAGACTTTCAGAAACCAAGCGGAGGGCTTTAAGAACGAGTCCAGTACCAACGCTACGAACACAAAGCAGTGGCGCGATGAGACCAAGGGTTTCCGAGACGAAGCCAAGCGGTTCAAGAATATGGCTGGTc                                                                                                                    >  1:258341/1‑301 (MQ=255)`  
                                                                                                                                                                                         `cTATCGGTGTCAATAACGATGGTCACTTGGATGCTCGTGGTCGTCGAATTGTGAACCTAGCGAACGCCGTGGATGACCGCGATGCTGTTCCGTTTGGTCAACTAAAGACCATGGACCAGAACTCATGGCAAGCACGTAATGAAGCCTTACAGTTCCGTAATGAGGCTGAGACTTTCAGAAACCAAGAGGAGGGCTTTAAGAACGAGTCCAGTACCAACGCGACGAACACAAAGCAGTGGCGCGATGAGACCAAGGGTTTCCGAGACGAAGCCGAGCGGTTCAAGAATACGGCTGGTCAATa                                                                                                                >  1:239249/1‑301 (MQ=255)`  
                                                                                                                                                                                         `cTATCGGTGTCAATAACGATGGTCACTTGGATGCTCGGGGTCGTCGAATTGTGAACCTAGCGAACGCCGTGGATGACCGCGATGCTGTTACGTTTGGTCAACTAAAGACCATGGACCAGAACTCATGGCAAGCACGTAATGAAGCCTTACAGTTCCGTAATGAGGCTGAGACTTTCAGAAACCAAGCGGAGGGCTTTAAGAACGAGTACAGTACCAACGCTACGAACACAAAGCAGTGGCGCGAAGAGACCAAGGGTATACGAAAAGAAGCCAAGCGGTTCAAGAATACGGCTGGTCAATa                                                                                                                >  1:150065/1‑301 (MQ=255)`  
                                                                                                                                                                                               `gtgtCAATAACGATGGTCACTTGGATGCTCGTGGTCGTCGAATTGTGAACCTAGCGAACGCCGTGGATGACCGCGATGCTGTTCCGTTTGGTCAACTAAAGACCATGGACCAGAACTCATGGCAAGCACGTAATGAAGCCTTACAGTTCCGTAATGAGGCTGAGACTTTCAGAAACCAAGCGGAGGGCTTTAAGAACGAGTCCAGTACCAACGCTACGAACACAAAGCAGTGGCGCGATGAGACCAAGGGTTTCCGAGACGAAGCCGAGCGGTTCAAGAATACGGCTGGTCAATACGCTAc                                                                                                          >  1:251199/1‑301 (MQ=255)`  
                                                                                                                                                                                               `gtgtCAATAACGATGGTCACTTGGATGCTCGTGGTCGTCGAATTGTGAACCTAGCGAACGCCGTGGATGACCGCGATGCTGTTCCGTTTGGTCAACTAAAGACCATGGACCAGAACTCATGGCAAGCACGTAATGAAGCCTTACAGTTCCGTAATGAGGCTGAGACTTTCAGAAACCAAGCGGAGGGCTTTAAGAACGAGTCCAGTACCAACGCTACGAACACAAAGCAGTGGCGCGATGAGACCAAGGGTTTCCGAGACGAAGCCAAGCGGTTCAAGAATACGGCTGGTCAATACGCTAc                                                                                                          <  1:254711/301‑1 (MQ=255)`  
                                                                                                                                                                                               `gagacagtAACGATGGTCACTTGGATGCTCGTGGTCGTCGAATTGTGAACCTAGCGAACGCCGTGGATGACCGCGATGCTGTTCCGTTTGGTCAACTAAAGACCATGGACCAGAACTCATGGCAAGCACGTAATGAAGCCTTACAGTTCCGTAATGAGGCTGAGACTTTCAGAAACCAAGCGGAGGGCTTTAAGAACGAGTCCAGTACCAACGCTACGAACACAAAGCAGTGGCGCGATGAGACCAAGGGTTTCCGAGACGAAGCCGAGCGGTTCAAGAATACGGCTGGTCAATACGCTAc                                                                                                          <  1:323932/294‑1 (MQ=255)`  
                                                                                                                                                                                                 `gtCAATAACGATGGTCACTTGGATGCTCGTGGTCGTCGAATTGTGAACCTAGCGAACGCCGTGGATGACCGCGATGCTGTTCCGTTTGGTCAACTAAAGAACATGGACCAGAACTCATGGCAAGCACGTAATGAAGCCTTACAGTTCCGTAATGAGGCTGAGACTTTCAGAAACCAAGCGGAGGGCTTTAAGAACGAGTCCAGTACCAACGCTACGAACACAAAGCAGTGGCACGATGAGACCAAGGGTTTCCGAGACGAAGCCGAGCGGTTCAAGaacagtctcttatacacatctccga                                                                                                        >  1:271296/1‑278 (MQ=255)`  
                                                                                                                                                                                                  `tCAATAACGATGGTCACTTGGATGCTCGTGGTCGTCGAATTGTGAACCTAGCGAACGCCGTGGATGACCGCGATGCTGTTCCGTTTGGTCAACTAAAGACCATGGACCAGAACTCATGGCAAGCACGTAATGAAGCCTTACAGTTCCGTAATGAGGCTGAGACTTTTAGAAACCAAGCGGAGGGCTTTAAGAACGAGTCCAGTACCAACGCTACGAACACAAAGCAGTGGCGCGATGAGACCAAGGGTTTCCGAGACGAAGCCGAGCGGTTCAAGAATACGGCTGGTCAATACGCTACATc                                                                                                       <  1:285089/301‑1 (MQ=255)`  
                                                                                                                                                                                                  `tCAAGACCGATGGTCACTTGAATGCTGGTTGTTGTCCACCTGTTAACCTAACCGCCGGCGTGGTTGACCGCCATGCTGTTCCGGTTTGTCAACTAAAGCCCATGGACCACAACTCATGGCAATCACGTAATGAAGCCTTCCAATTCCGTAATGAGGCTGAGACTTTCAGAAACCAAACGGAAGGCTTTAAGAACGAGTCCAGTACCAACTCTACGAACACAAAGCTGTTGCGCGATGAGACCAAGGGTTTCCGAGACGAAGCCGAGCGGTTCAAGAATACGGCTGGTCAATACGCTACATc                                                                                                       <  1:100204/301‑1 (MQ=255)`  
                                                                                                                                                                                                   `cAATAACGATGGTCACTTGGATGCTCGTGGTCGTCGAATTGTGAACCTAGCGAACGCCGTTGATGACCGCGATGCTGTTCCGTTTGGTCAACTAAAGACCATGGACCAGAACTCATGGCAAGCACGTAATGAAGCCTTACAGTTCCGTAATGAGGCTGAGACTTTCAGAAACCAAGCGGAGGGCTTTAAGAACTAGTCCAGTACCAACGCTACGAACACAAAGCAGTGGCGCGATGAGACCAAGGGTTTCCGAGACGAAGCCAAGCGGTTCAAGAATACGGcctgtctcttatacacatct                                                                                                      >  1:268673/1‑282 (MQ=255)`  
                                                                                                                                                                                                       `aaCGATGGTCACTTGGATGCTAGTGGTCGTCGAATTGTGAACCTAGCGAACGCCGTGGATGACCGCGATGCTGTTCCGTTTGGTCAACTAAAGACCATGGACCAGAACTCATGGCAAGCACGTAATGAAGCCTTACAGTTCCGTAATGAGGCTGAGACTTTCAGAAACCAAGCGGAGGGCTTTAAGAACGAGTCCAGTACCAACGCTACGAACACAAAGCAGTGGCGCGATGAGACCAAGGGTTTCCGAGACGAAGCCAAGCGGTTCAAGAATACGGCTGGTCAATACGCTACATctactg                                                                                                  <  1:21117/301‑1 (MQ=255)`  
                                                                                                                                                                                                        `aCGATGGTCACTTGGATGCTCGTGGTCGTCGAATTGTGAACCTAGCGAACGCCGTGGATGACCGCGATGCTGTTCCGTTTGGTCAACTAAAGACCATGGACCAGAACTCATGGCAAGCACGTAATGAAGCCTTACAGTTCCGTAATGAGGCTGAGACTTTCAGAAACCAAGCGGAGGGCTTTAAGAACGAGTCCAGTACCAACGCTACTAACACAAAGCAGTGGCGCGATGAGACCAAGGGTTTCCGAGACGAAGCCAAGCGGGTCAAGaactgtctcttatacacatctccgagcccacg                                                                                                 >  1:234180/1‑271 (MQ=255)`  
                                                                                                                                                                                                        `aCGATGGTCACTTGGATGCTCGTGGTCGTCGAATTGTGAACCTAGCGAACGCCGTGGATGACCGCGATGCTGTTCCGTTTGGTCAACTAAAGACCATGGACCAGAACTCATGGCAAGCACGTAATGAAGCCTTACAGTTCCGTAATGAGGCTGAGACTTTCAGAAACCAAGCGGAGGGCTTTAAGAACGAGTCCAGTACCAACGCTACGAACACAAAGCAGTGGCGCGATGAGACCAAGGGTTTCCGAGACGAAGCCAAGCGGTTCAAGAATACGGCTGGTCAATACGCTACATCTGCTgg                                                                                                 >  1:165390/1‑301 (MQ=255)`  
                                                                                                                                                                                                        `aCGATGGTCACTTGGATGCTCGTGGTCGTCGAATTGTGAACCTAGCGAACGCCGTGGATGACCGCGATGCTGTTCCGTTCGGTCAACTAAAGACCATG‑GCCAGAACTCATGGCAAGCACGTAATGAAGCCTTACAGTTCCGTAATGAGGCTGAGACTTTCAGAAACCAAGCGGAGGGCTTTAAGAACGAGTCCAGTACCAACGCTACGAACACAAAGCAGTGGCGCGATGAGACCAAGGGTTTCCGAGACGAAACCAAGCGGTTCAAGAATACGGCTGGTTAATAAGCTCCATCTGCTggg                                                                                                >  1:16973/1‑301 (MQ=255)`  
                                                                                                                                                                                                            `tGGTCACTGGTATGCTCGTGGTAGTCGAATTGTGAACCTAGCGAAAGCCGTGGATGACCGCGATGCTGTTCCGTTTGGTCAACTAAAGACCATGGACCAGAACGCATGGCAAGCACGTAATGAAGCCGTACAGTTCCGTAATGAGGCTGAGACTTTCAGAAACCAAGCGGAGGGCTTTAAGAACGAGTCCAGTACCAACGCTACGAACACAAAGCAGTGGCGCGATGAGACCAAGGGTTTCCGAGACGAAGCCAAGCGGTTCAAGAATACGGCTGGTAAATACGCTACATCTGCTGGGAAc                                                                                             <  1:91018/301‑1 (MQ=255)`  
                                                                                                                                                                                                                       `aTGCTCGTGGTCGTCGAATTGTGAAACTAGCGAACGCCGTGGATGACCGCGATGCTGTTCCGTTTGGTCAACTAAAGACCATGGACAAGAACTCATGGCAAGCACGTAATGAAGCCTTACAGTTCCGTAATGAGGCTGAGACTTTCAGAAACCAAGCAGAGGGCTTTAAGAACGAGTCCAGTACCAACGCTACGAACACAAAGCAGTGGCGCGATGAGACCAAGGGTTTCCGAGACGAAGCCGAGCGGTTCAAGAATACGGCTGGTCAATACGCTACATCTGCTGGGAACTCTGCTTCCGc                                                                                  <  1:311097/301‑1 (MQ=255)`  
                                                                                                                                                                                                                                    `tcgAATTGTGAACCTAGCGAACGCCGTGGATGACCGCGATGCTGTTCCGTTTGGTCAACTAAAGACCATGGACCAGAACTCATGGCAAGCACGTAATGAAGCCTTACAGTTCCGTAATGAGGCTGAGACTTTCAGAAACCAAGCGGAGGGCTTTAAGAACGAGTCCAGTACCAACGCTACGAACACAAAGCAGTGGCGCGATGAGACCAAGGGTTTCCGAGACGAAGCCGAGCGGTTCAAGAATACGGCTGGTCAATACGCTACATCTGCTCGGAACTCTGCTTCCGCTGCGCATCAATCt                                                                     <  1:237025/301‑1 (MQ=255)`  
                                                                                                                                                                                                                                    `tcgAATTGTGAACCTAACGAACGCCGTGGATGACCGCGATGCTGTTCCGTTTGGTCAACTAAAGACCATGGACCAGAACTCATGGCAAGCACGTAATGAAGCCTTACAGTTCCGTAATGAGGCTGAGACTTTCAGAAACCAAGCGGAGGGCTTTAAGAACGAGTCCAGTACCAACGCTACGAACACAAAGCAGTGGCGCGATGAGACCAAGGGTTTCCGAGACGAAGCCAAGCGGTTCAAGAATACGGCTGGTCAATACGCTACATCTGCTGGGAACTCTGCTTCCGCTGCGCATCAATCt                                                                     >  1:265568/1‑301 (MQ=255)`  
                                                                                                                                                                                                                                        `aTTGTGAACCTAGCGAACGCCGTGGATGACCGCGATGCTGTTCCGTTTGGTCAACTAAAGACCATGGACCAGAACTCATGGCAAGCACGTAATGAAGCCTTACAGTTCCGTAATGAGGCTGAGACTTTCAGAAACAAAGCGGAGGGCTTTAAGAACGAGTCCAGTACCAACGCTACGAACACAAAGCAGTGGCGAGATGAGACCAAGGGTTTCCGAGACGAAGCCAAGCGGTTCAAGAATATGGCTGGTCAATACGCTACATCTGCTGGGAACTCTGCTTCCGCTACGCATctgtctcttt                                                                 >  1:65193/1‑292 (MQ=255)`  
                                                                                                                                                                                                                                         `ttGTGAACCTAGCGAACGCCGTGGATGACCGCGATGCTGTTCCGTTTGGTCAACTAAAGACCATGGACCAGAACTCATGGCAAGCACGTAATGAAGCCTTACAGTTCCGTAATGAGGCTGAGACTTTCAGAAACCAAGCGGAGGGCTTTAAGAACGAGTCCAGTACCAACGCTACGAACACAAAGCAGTGGCGCGATGAGACCAAGGGTTTCCGAGACGAAGCCGAGCGGTTCAAGAATACGGCTGGTCAATACGCTACATCTGCTGGGAACTCTGCTTCCGCTGCGCATCAATCTGAGGt                                                                <  1:247699/301‑1 (MQ=255)`  
                                                                                                                                                                                                                                           `gtgAACCTAGCGAACGCCGTGGATGACCGCGATGCTGTTCCGTTTGGTCAACTAAAGACCATGGACCAGAACTCATGGCAAGCACGTAATGAAGCCTTACAGTTCCGTAATGAGGCTGAGACTTTCAGAAACCAAGCGGAGGGCTTTAAGAACGAGTCCAGTACCAACGCTACGAACACAAAGCAGTGGCGCGATGAGACCAAGGGTTTCCGAGACGAAGCCAAGCGGTTCAAGAATACGGCTGGTCAATACGCTACATCTGCTGGGAACTCTGCTTCCGCTGCGCATCAATCTGAGGTaa                                                              >  1:334885/1‑301 (MQ=255)`  
                                                                                                                                                                                                                                                   `aGCGAACGCCGTGGATGACCGCGATGCTGTTCCGTTTGGTCAACTAAAGACCATGGACCAGAACTCATGGCAAGCACGTAATGAAGCCTTACAGTTCCGTAATGAGGCTGAGACTTTCAGAAACCAAGCGGAGGGCTTTAAGAACGAGTCCAGTACCAACGCTACGAACACAAAGCAGTGGCGCGATGAGACCAAGGGTTTCCGAGACGAAGCCAAGCGGTTCAAGAATATGGCTGGTCAATACGCTACATCTGCTGGGAACTCTGCTTCCGCTGCGCATCAATCTGAGGTAAACGCTgag                                                      >  1:55889/1‑301 (MQ=255)`  
                                                                                                                                                                                                                                                      `gAACGCCGTGGATGACCGCGATGCTGTTCCGTTTGGTCAACTAAAGACCATGGACCAGAACTCATGGCAAGCACGTAATGAAGCCTTACAGTTCCGTAATGAGGCTGAGACTTTCAGAAACCAAGCGGAGGGCTTTAAGAACGAGTCCAGTACCAACGCTACGAACACAAAGCAGTGGCGCGATGAGACCAAGGGTTTCCGAGACGAAGCCGAGCGGTTCAAGAATACGGCTGGTCAATACGCTACATCTGCTGGGAACTCTGCTTCCGCTGCGCATCAATCTGAGGTAAACGCTGAGAAc                                                   <  1:141908/301‑1 (MQ=255)`  
                                                                                                                                                                                                                                                       `aaCGCCGTGGATGACCGCGATGCTGTTCCGTTTGGTCAACTAAAGACCATGGACCAGAACTCATGGCAAGCACGTAATGAAGCCTTACAGTTCCGTAATGAGGCTGAGACTTTCAGAAACCAAGCGGAGGGCTTTAAGAACGAGTCCAGTACCAACGCTACGAACACAAAGCAGTGGCGCGATGAGACCAAGGGTTTCCGAGACGAAGCCGAGCGGTTCAAGAATACGGCTGGTAAATACGCTACATCTGCTGGGAACTCTGCTTCCGCTGCGCATCAATCTGAGGTAAACGCTGAGAAct                                                  <  1:275082/301‑1 (MQ=255)`  
                                                                                                                                                                                                                                                         `cGCCGTGGATGACCGCGATGCTGTTCCGTTTGGTCAACTAAAGACCATGGACCAGAACTCATGGCAAGCACGTAATGAAGCCTTACAGTTCCGTAATGAGGCTGAGACTTTCAGAAACCAAGCGGAAGGCTTTAAGAACGAGTCCAGTACCAACGCTACGAACACAAAGCAGTGGCGCGATGAGACCAAGGGTTTCCGAGACGAAGCCGAGCGGTTCAAGAATACGGCTGGTCAATACGCTACATCTGCTGGGAACTCTGCTTCCGCTGCGAATCAATCTGAGGTAAACGCTGAGAAcctg                                                >  1:129916/1‑298 (MQ=255)`  
                                                                                                                                                                                                                                                           `ccGTGGATGACCGCGATGCTGTTCCGTTTGGTCAACTAAAGACCATGGACCAGAACTCATGGCAAGCACGTAATGAAGCCTTACAGTTCCGTAATGATGCTGAGACTTTCAGAAACCAAGCGGAGGGCTTTAAGAACTAGTCCAGTACCAACGCTACGAACACAAAGCAGTGGCGCTATGAGACCAAGGGTTTCCGAGACGAAGCCGAGCGGTTCAAGAATACGGCTGGTCAATACGCTACATCTGCTGGGAACTCTGCTTCCGCTGCGCATCAATCTTAGGTAAACGCTGAGAACTCTGc                                              >  1:120659/1‑301 (MQ=255)`  
                                                                                                                                                                                                                                                           `ccGTGGATGACCGCGATGCTGTTCCGTTTGGTCAACTAAAGACCATGGACCAGAACTCATGGCAAGCACGTAATGAAGCCTTACAGTTCCGTAATGAGGCTGAGACTTTCAGAAACCAAGCGGAGGGCTTTAAGAACGAGTCCAGTACCAACGCTACGAACACAAAGCAGTGGCGCGATGAGACCAAGGGTTTCCGAGACGAAGCCAAGCGGTTCAAGAATACGGCTGGTCAATACGCTACATCTGCTGGGAACTCTGCTTCCGCTGCGCATCAATCTGAGGTAAACGCTGAGAACTCTGc                                              <  1:282424/301‑1 (MQ=255)`  
                                                                                                                                                                                                                                                               `ggATGACCGCGATGCTGTTCCGTTTGGTCAACTAAAGACCATGGACCAGAACTCATGGCAAGCACGTAATGAAGCCTTACAGTTCCGTAATGAGGCTGAGACTTTCAGAAACCAAGCGGAGGGCTTTAAGAACGAGTCCAGTACCAACGCTACGAACACAAAGCAGTGGCGCGATGAGACCAAGGGTTTCCGAGACGAAGCCGAGCGGTTCAAGAATACGGCTGGTCAATACGCTACATCTGCTGGGAACTCTGCTTCCGCTGCGCATCCATCTGAGGTAAACGCTGAGAACTATGCcacc                                          >  1:340275/1‑300 (MQ=255)`  
                                                                                                                                                                                                                                                                `aaagagacagATGCTGTTCCGTTTGGTCAACTAAAGACCATGGACCAGAACTCATGGCAAGCACGTAATGAAGCCTTACAGTTCCGTAATGAGGCTGAGACTTTCAGAAACCAAGCGGAGGGCTTTAAGAACGAGTCCAGTACCAACGCTACGAACACAAAGCAGTGGCGCGATGAGACCAAGGGTTTCCAAGACGAAGCCAAGCGGTTCAAGAATACGGCTGGTCAATACGCTACATCTGCTGGGAACTCTGCTTCCGCTGCGCATCAATCTGAGGTAAACGCTGAGAACTCTGCCACAg                                         <  1:109044/292‑1 (MQ=255)`  
                                                                                                                                                                                                                                                                           `tGCTGTTCCGTTTTGTCAACTAAAGACCATGGACCAGAACTCATGGCAAGCACGTACTGAAGCCTTACAGTTCCGTAATGAGGCTGAGACTTTCAGAAACCAAGCGGAGGGCTTTAAGAACGAGTCCAGTACAAACGCTACGAACACAAAGCAGTGGCGCGATGAGACCAAGGGTTTCCGAGACGAAGCCGAGCGGTTCAAGAATACGGCTGGTCAATACGCTACATCTGCTGGGAACTCTGCTTCCGCTGCGCATCAATCTGAGGTAAACGCTGAGAACTCTGCCACAGCATCCGCTAAc                              <  1:176414/301‑1 (MQ=255)`  
                                                                                                                                                                                                                                                                           `gatgtgtataagagacagaCTAAAGACCATGGACCAGAACTCATGGCAAGCACGTAATGAAGCCTTACAGTTCCGTAATGAGGCTGAGACTTTCAGAAACCAAGCGGAGGGCTTTAAGAACGAGTCCAGTACCAACGCTACGAACACAAAGCAGTGGCGCGATGAGACCAAGGGTTTCCGAGACGAAGCCGAGCGGTTCAAGAATACGGCTGGTCAATACGCTACATCTGCTGGGAACTCTGCTTCCGCTGCGCATCAATCTGAGGTAAACGCTGAGAACTCTGCCACAGCATCCGCTAAc                              <  1:144069/283‑1 (MQ=255)`  
                                                                                                                                                                                                                                                                             `tgtgtataagagaaagaCTAAAGACCATGGACCAGAACTCATGGCAAGCACGTAATGAAGCCTTACAGTTCCGTAATGAGGCTGAGACTTTCAGAAACCAAGCGGAGGGCTTTAAGAACGAGTCCAGTACCAAAGCTACGAACACAAAGCAGTGGCGCGATGAGACCAAGGGTTTCCGAGACGAAGCCGAGCGGTTCAAGAATACGGCTGGTCAATACGCTACATCTGCTGGGAACTCTGCGTCCGCTGCGCATCAATCTGAGGTAAACGCTGAGAACTCTGCCACAGCATCCGCTAActa                            <  1:199258/285‑2 (MQ=255)`  
                                                                                                                                                                                                                                                                              `tGTTCCGTTTGGTCAACTAAAGACCATGGACCAGAACTCATGGCAAGCACGTAATGAAGCCTTACAGTTCCGTAATGAGGCTGAGACTTTCAGAAACCAAGCGGAGGGCTTTAAGAACGAGTCCAGTACCAACGCTACGAACACAAAGCAGTGGCGCGATGAGACCAAGGGTTTCCGAGACGAAGCCAAGCGGTTCAAGGATACGGCTGGTAAATACGCTACATCTGCTGGGAACTCTGCTTCCGCTGCGCATCAATCTGAGGTAAACGCTGAGAACTCTGCCACAGCATCCGCTAActat                           >  1:215250/1‑299 (MQ=255)`  
                                                                                                                                                                                                                                                                               `gTTCCGTTTGGTCAACTAAAGACCATGGACCGGAACTCATGGCAAGCACGTAATGAAGCCTTACAGTTCCGTAATGAGGCTGAGACTTTCAGAAACCAAGCGGAGGGCTTTAAGAACGAGTCCAGTACAAACGCTACGAACACAAAGCAGTGGCGCGATGAGACCAAGGGTTTCCGAGACGAAGCCAAGCGGTTCAAGAATATGGCTGGTCAATACGCTACATCTGCTGGGAACTCTGCTTCCGCTGCGCATCAATCTGAGGTAAACGCTGAGAACTCTGCCACAGCATCCGCTAActatt                          >  1:242822/1‑298 (MQ=255)`  
                                                                                                                                                                                                                                                                                  `ccGTTTGGTCAACTAAAGACCATGGACCAGAACTCATGGCAAGCACGTAATGAAGCCTTACAGTTCCGTAATGAGGCTGAGACTTTCAGAAACCAAGCGGAGGGCTTTAAGAACGAGTCCAGTACCAACGCTACGAACACAAAGCAGTGGCGCGATGAGACCAAGGGTTTCCGAGACGAAGCCAAGCGGTTCAAGAATACTGCTGGTCAATACGCTACATCTGCTGGGAACTCTGCTTCCGCTGCGCATCAATCTGAGGTAAACGCTGAGAACTCTGCCACAGCATCCGCTAACTATGCTc                       >  1:32714/1‑301 (MQ=255)`  
                                                                                                                                                                                                                                                                                   `cGTTTGGTCAACTAAAGACCATGGACCAGAACTCATGGCAAGCACGTAATGAAGCCTTACAGTTCCGTAATGAGGCTGAGACTTTCAGAAACCAAGCGGAGGGCTTTAAGAACGAGTCCAGTACCAACGCTACGAACACAAAGCAGTGGCGCGATGAGACCAAGGGTTTCCGAGACGAAGCCAAGCGGTTCAAGAATACGGCTGGTCAATACGCTACATCTGCTGGGAACTCTGCTTCCGCTGCGCATCAATCTGAGGTAAACGCTGAGAACTCTGCCACAGCATCCGCTAACTATGCTCa                      <  1:287950/301‑1 (MQ=255)`  
                                                                                                                                                                                                                                                                                       `tGGTCAACTAAAGACCATGGACCAGAACTCATGGCAAGCACGTAATGAAGCCTTACAGTTCCGTAATGAGGCTGAGACTTTCAGAAACCAAGCGGAGGGCTTTAAGAACGAGTCCAGTACCAACGCTACGAACACAAAGCAGTGGCGCGATGAGACCAAGGGTTTCCGAGACGAAGCCGAGCGGTTCAAGAATACGGCTGGTCAATACGCTACATCTGCTGGGAACTCTGCTTCCGCTGCGCATCAATCTTAGGTAAACGCTGAGAACTCTGCCACAGCATCCGCTAACTCTGCTCATTTg                  <  1:153632/301‑1 (MQ=255)`  
                                                                                                                                                                                                                                                                                             `aCTAAAGACCATGGACCAGAACTCATGGCAAGCACGTAATGAAGCCTTACAGTTCCGTAATGAGGCTGAGACTTTCAGAAACCAAGCGGAGGGCTTTAAGAACGAGTCCAGTACCAACGCTACGAACACAAAGCAGTGGCGCGATGAGACCAAGGGTTTCCGAGACGAAGCCGAGCGGTTCAAGAATACGGCTGGTCAATACGCTACATCTGCTGGGAACTCTGCTTCCGCTGCGCATCAATCTGAGGTAAACGCTGAGAACTCTGCCACAGCATCCGCTAAccactgtctcttatacaca            >  1:53238/1‑283 (MQ=255)`  
                                                                                                                                                                                                                                                                                                `aaaGACCATGAACCAGAACTCATGGCAAGCACGTAATGAAGCCTTACAGTTCCGTAATGAGGCTGAGACTTTCAGAAACCAAGCGGAGGGCTTTAAGAACGAGTCCAGTACCAACGCTACGAACACAAAGCAGTGGCGCGATGAGACCAAGGGTTTCCGAGACGAAGCCGAGCGGTTCAAGAATACGGCTGGTCAATACGCTACATCTGCTGGGAACTCTGCTTCCGCTGCGCATCAAGCTGAGGTAAACGCTGAGAACTCTGCCACAGCATCCGCTAACTATGCTCATTTGGCAGAACag         >  1:118755/1‑301 (MQ=255)`  
                                                                                                                                                                                                                                                                                                     `ccATGGACCAGAACTCATGGCAAGCACGTAATGAAGCCTTACAGTTCCGAAATGAGGCTGAGACTTTCAGAAACCAAGCGGAGGGCTTTAAGAACGAGTCCAGTACCAACGCTATGAACACAAAGCAGTGGCGCGATGAGACCAAGGGTTTCCGAGACGAAGCTGAGCGGTTCAAGAATACGGCTGGTCAATACGCTACATCTGCTAGGAACTCTGCTTCCGCTGCGCATCAATCTGAGGTAAACGCTGAGAACTCTGCCACAGCATCCGCTAACTCTGCTCATTTGGCAGAACagcaagc    <  1:140118/301‑1 (MQ=255)`  

                                                                                                                                                                                                                                                                                                          `|`                                                                                                                                                                                                                                                                                                           
`CTCTCTGACAAAGGCTTGGGGTCCAGCCGATGGCTACACGACCATCGAGTTACGTCGAGTAACCTCCACTACCGACCGATTGGTTGACTTTACGGATGGTTCAATCCTCCGCGCGTATGACCTTAACGTCGCTCAGATTCAAACGATGCACGTAGCGGAAGAGGCCCGTGACCTCACTACGGATACTATCGGTGTCAATAACGATGGTCACTTGGATGCTCGTGGTCGTCGAATTGTGAACCTAGCGAACGCCGTGGATGACCGCGATGCTGTTCCGTTTGGTCAACTAAAGACCATGAACCAGAACTCATGGCAAGCACGTAATGAAGCCTTACAGTTCCGTAATGAGGCTGAGACTTTCAGAAACCAAGCGGAGGGCTTTAAGAACGAGTCCAGTACCAACGCTACGAACACAAAGCAGTGGCGCGATGAGACCAAGGGTTTCCGAGACGAAGCCAAGCGGTTCAAGAATACGGCTGGTCAATACGCTACATCTGCTGGGAACTCTGCTTCCGCTGCGCATCAATCTGAGGTAAACGCTGAGAACTCTGCCACAGCATCCGCTAACTCTGCTCATTTGGCAGAACAGCAAGCAG  >  NC_001604.1/34797‑35392`

  

Alignment Legend

Aligned base mismatch/match (shaded by quality score): `ATCG/ATCG` `< 3 ≤` `ATCG/ATCG` `< 10 ≤` `ATCG/ATCG` `< 22 ≤` `ATCG/ATCG` `< 34 ≤` `ATCG/ATCG` `< 38 ≤` `ATCG/ATCG`

Unaligned base: `atcg`    Masked matching base: `atcg`    Alignment gap: `‑`    Deleted base: `‑`
